# Zerumbone Restores Gut Microbiota Composition in ETBF Colonized AOM/DSS Mice

**DOI:** 10.4014/jmb.2006.06034

**Published:** 2020-09-14

**Authors:** Hye-Won Cho, Ki-Jong Rhee, Yong-Bin Eom

**Affiliations:** 1 Department of Medical Sciences, College of Medical Sciences, Soonchunhyang University, Asan 3538, Republic of Korea; 2 Department of Biomedical Laboratory Science, College of Health Sciences, Yonsei University at Wonju, Wonju, 6493, Republic of Korea; 3 Department of Biomedical Laboratory Science, College of Medical Sciences, Soonchunhyang University, Asan 158, Republic of Korea

**Keywords:** Colorectal cancer, ETBF, inflammation, microbial diversity, zerumbone

## Abstract

Colorectal cancer (CRC) is the leading cause of common malignant neoplasm worldwide. Many studies have analyzed compositions of gut microbiota associated with various diseases such as inflammatory bowel diseases (IBD) and colon cancer. One of the most representative bacteria involved in CRC is enterotoxigenic *Bacteroides fragilis* (ETBF), a species belonging to phylum Bacteroidetes. We used ETBF colonized mice with azoxymethane (AOM)/dextran sulphate sodium (DSS) and zerumbone, a compound with anti-bacterial effect, to determine whether zerumbone could restore intestinal microbiota composition. Four experimental groups of mice were used: sham, ETBF colonized AOM/DSS group, ETBF colonized AOM/DSS group zerumbone 60 mg kg^-1^ (ETBF/AOM/ DSS + Z (60)), and only zerumbone (60 mg kg^-1^)-treated group. We performed reversible dye terminators-based analysis of 16S rRNA gene region V3-V4 for group comparison. Microbiota compositions of ETBF/AOM/DSS + Z (60) group and ETBF colonized AOM/DSS group not given zerumbone were significantly different. There were more *Bacteroides* in ETBF/AOM/DSS + Z (60) group than those in ETBF colonized AOM/DSS group, suggesting that *B. fragilis* could be a normal flora activated by zerumbone. In addition, based on linear discriminant analysis of effect size (LEfSe) analysis, microbial diversity decreased significantly in the ETBF colonized AOM/DSS group. However, after given zerumbone, the taxonomic relative abundance was increased. These findings suggest that zerumbone not only influenced the microbial diversity and richness, but also could be helpful for enhancing the balance of gut microbial composition. In this work, we demonstrate that zerumbone could restore the composition of intestinal microbiota.

## Introduction

Colorectal cancer (CRC) is the third most common cancer and the fourth leading cause of common malignant neoplasm worldwide, accounting for approximately 1.2 million new cases and 600,000 deaths per year [1, 2], The common use of fecal samples to study microbial composition may reflect disease state [3]. With advance in metagenomic technology, growing evidence now suggests that dysbiosis, *i.e.*, disorder of normal intestinal microbiota, can promote inflammatory conditions and production of carcinogenic metabolites [4, 5].

The gut commensal microbiota as a mutualistic ecosystem plays vital roles in maintaining host health such as digestion of dietary components, production of metabolites (fatty acids, vitamins), and inducing disease [6-8]. Many studies have analyzed compositions of intestinal microbes to identify various disorders such as inflammatory bowel diseases (IBD), colon cancer, obesity, and diabetes [9-11]. The most abundantly phylum found in the body include Firmicutes, Bacteroidetes, Actinobacteria, and Proteobacteria [12]. Among these phyla, we have used *Bacteroides fragilis* (*B. fragilis*), a species belonging to phylum Bacteroidetes, and zerumbone, a compound with anti-bacterial and anti-biofilm effect on *B. fragilis* [13, 14], to demonstrate that zerumbone could balance the composition of intestinal microbiota in microbial dysbiosis. Gram-negative *Bacteroides* species are most dominant constituents of the gut microbiota [15]. As a representative species belonging to *Bacteroides*, *B. fragilis* is divided into two subtypes: non-enterotoxigenic *B. fragilis* (NTBF) and enterotoxigenic *B. fragilis* (ETBF). Recently, non-toxigenic *B. fragilis* (NTBF) has been shown to have health beneﬁts for the host. It is regarded as a probiotic candidate [16-18]. Wang *et al*. have suggested that *B. fragilis* will be the ﬁrst probiotic species from the phylum Bacteroidetes [19].

Medical herbs and plant foods contain many biologically active phytochemicals that have various health-promoting effects [20]. Zerumbone is a main component of the edible ginger plant *Zingiber zerumbet*, originating in South-East Asia, and has been cultivated for thousands of years as a spice and for medical purposes [21]. *Zingiber zerumbet* contains several types of phytochemical and is considered as one of the most generally used traditional dietary seasoning throughout Asia [22].

Recent studies revealed several biological properties of zerumbone that may be responsible for inhibition of ETBF-induced colon carcinogenesis. Zerumbone has been reported to have anti-inflammatory and anti-cancer activities [23-26]. Our previous study demonstrated that zerumbone has anti-biofilm and antimicrobial effects against ETBF [14]. Apparently, it is hypothesized that intake of zerumbone may result in enhanced protection from the development of microbial dysbiosis associated with ETBF. However, to the best of our knowledge, no studies have reported preventive or balancing effects of zerumbone on ETBF-mediated dysbiosis in mice.

The purpose of this study was to determine whether oral treatment of zerumbone might have the ability to alter the composition of intestinal microbiota in mice with CRC caused by ETBF. Here, we assessed whether the zerumbone-induced improvements in gut microbiota in mice colonized with ETBF using the Illumina MiSeq platform.

## Materials and Methods

### Bacteriology

The wild-type ETBF strain used in our study was *B. fragilis* 86-5443-2-2 (*bft-2*). This wild-type *Bacteroides* strain was resistant to gentamicin and clindamycin. Colonization of bacteria was monitored by serial dilution and plating of stool on brain heart infusion agar plates containing 50 μg ml^-1^ of gentamicin (Corning, USA) and 6μg ml^-1^ of clindamycin (Hospira, USA). Characteristic *B. fragilis* colonies were enumerated after anaerobic culture and shown as colony-forming units (CFU) gram-1 stool. The bacterial strain was a generous gift from Cynthia Sears and Augusto Franco-Mora (Johns Hopkins University, USA).

### ETBF/AOM/DSS Mouse Model

The experimental protocol was approved by the Institutional Animal Care and Use Committee of Yonsei University at Wonju, Korea (approval number: YWCL-201901-002-01). All experiments were performed in accordance with relevant guidelines under the Institutional Animal Care and Use Committee of Yonsei University at Wonju, Korea. Female BALB/c mice at 8-week-old (Rion-bio, Republic of Korea) received a single intraperitoneal injection of 10 mg kg^-1^ of AOM (Sigma-Aldrich, USA). ETBF/AOM/DSS mouse model was constructed as described previously [26, 27] with minor modifications. In brief, at two days after AOM injection, mice were given water containing clindamycin (100 mg l^-1^) and gentamicin (300 mg l^-1^) for 5 days to promote colonization of *B. fragilis*. Mice were inoculated with bacteria. Antibiotic-containing water was continued for additional 7 days. After 7 days of distilled water (DW), mice were administrated with the first DSS cycle (5 days DSS, 16 days of DW) for a total of 3 cycles. DSS (36-50 kDa) was purchased from MP Biomedicals (USA). Bacteria were grown in brain heart infusion broth (Becton, Dickinson and Company, USA) and adjusted to 1 × 10^9^ CFU 200 µl^-1^ for mouse oral inoculations.

### Zerumbone Treatment and DNA Extraction

Zerumbone was purchased from Kingherbs (China). BALB/c mice were given AOM once (10 mg kg^-1^), ETBF (1 × 10^9^ CFU 200 µl^-1^), and 2 cycles of DSS (1%). Administration of zerumbone was initiated simultaneously with DSS treatment. During 2 cycles of DSS, BALB/c mice were treated with zerumbone (60 mg kg^-1^, p.o.) three times a week. Mice from each group were monitored daily for water consumption. Cecum contents were stored at −80°C immediately after sample collection. DNA was extracted from all samples using FastDNA SPIN Kit for feces (MPbio, USA) according to the manufacturer's instructions and stored at −80°C prior to amplification.

### PCR Amplification and MiSeq Sequencing

PCR was performed for the 16S rRNA hypervariable region V3-V4 using primers of 341F (5’-TCGTCGGCA GCGTCAGATGTGTATAAGAGACAG-CCTACGGGNGGCWGCAG-3’, underlining sequence indicating target region primer) and 805R (5’-GTCTCGTGGGCTCGG-AGATGTGTATAAGAGACAG-GACTACHVGGGTATCTAATCC-3’) as described previously [28]. PCR conditions were as follows: initial denaturation at 95°C for 3 min followed by 25 cycles of denaturation at 95°C for 30 sec, primer annealing at 55°C for 30 sec, and extension at 72°C for 30 sec, with a final elongation step at 72°C for 5 min. Secondary amplification for attaching Illumina NexTera barcode was then performed with i5 forward primer (5’-AATGATACGGCGACCACCGAGATCTACAC-XXXXXXXXTCGTCGGCAGCGTC-3’; X indicates the barcode region) and i7 reverse primer (5’CAAGCAGAA GACGGCATACGAGAT-XXXXXXXX-GTCTCGTGGGCTCGG-3’). The condition of secondary amplification was similar to the former one except that the amplification cycle was set to 8. The PCR product was confirmed by 1% agarose gel electrophoresis and visualized under a Gel Doc system (BioRad, Hercules, CA, USA). Amplified PCR products were purified with CleanPCR (CleanNA, Waddinxveen, Netherlands). Equal concentrations of purified products were pooled together and short fragments (non-target products) were removed with CleanPCR (CleanNA). The quality and product size were assessed on a Bioanalyzer 2100 (Agilent, USA) using a DNA 7500 chip. Mixed amplicons were pooled and sequencing was carried out at Chunlab, Inc. (Republic of Korea) with Illumina MiSeq Sequencing system (Illumina, USA) according to the manufacturer’s instructions.

### Microbiome Taxonomic Profiling

Processing raw reads starts with quality check and filtering the following leads, (1) Average quality less than 25 leads (AVGQUAL:25), (2) Lead length less than 100 (MINLEN:100) using the Trimmomatic program 0.32 [29]. For quality filtering, the adapter trimming work was performed separately with ChunLab’s in-house code using the merged sequence, and the average quality for each lead is calculated to remove the leads that under the standard. After QC pass, paired-end sequence data were merged together using PANDAseq [30]. Primers were then trimmed with ChunLab’s in-house program at a similarity cut off of 0.8. Nonspecific amplicons that did not encode 16S rRNA were detected by using HMMER’s hmmsearch program [31] with 16S rRNA profiles. Sequences were denoised using DUDE-Seq [32] and non-redundant reads were extracted by UCLUST-clustering [33]. EzBioCloud database was used for taxonomic assignment using USEARCH (8.1.1861_i86linux32) [33] followed by more precise pairwise alignment [34]. UCHIME and the non-chimeric 16S rRNA database from EzBioCloud were used to detect chimera on reads that contained less than 97% best hit similarity rate. Sequence data were then clustered using CD-HIT [35] and UCLUST and defined OTUs in this step.

### Statistical Analysis

Comparison of mean was performed using unpaired, two-tailed Mann-Whitney U test unless otherwise indicated. All statistical analyses were performed using GraphPad Prism (GraphPad Software Inc, USA) and EzBioCloud (ChunLab, Inc, Seoul, Korea). Statistical significance was set at *p*-value < 0.05. LEfSe analysis was used to predict how zerumbone treatment impact their composition of gut microbiome in the main functional levels (KEGG categories) [36]. LEfSe exploits the Kruskal–Wallis rank-sum test to identify significantly different characteristics between assigned taxa compared to the groups, and uses Linear discriminant analysis (LDA) to estimate the size effect, based on a LDA socre > 2.0 and *p*-value < 0.05. The LEfSe were analyzed by the EzBioCloud database (ChunLab, Inc.) [37]. Alpha diversity analysis expressed with ACE (http://www.mothur.org/wiki/Ace), Chao1 (http://www.mothur.org/wiki/Chao), Jackknife (http://www.mothur.org/wiki/Jack), OTUs, Shannon (http://www.mothur.org/wiki/Shannon) and Simpson (http://www.mothur.org/wiki/Simpson) were conducted using CLcommunity software (ChunLab, Inc.).

## Results

### Effects of Zerumbone on the Composition of Gut Microbiome at Phylum Levels

Microbial dysbiosis caused by inflammation and tumorigenesis directly contributes towards polyp formation in the AOM/DSS model [38]. It was hypothesized that zerumbone treatment led to a decrease in ETBF-mediated dysbiosis by altering microbial composition. Therefore, in order to test the hypothesis, we analyzed bacterial communities in all stool specimens to assess differences in the composition of gut microbiota between sham, ETBF colonized group, and zerumbone treated groups. 16S rRNA gene clone libraries were established and sequenced.

**Fig. 1:**
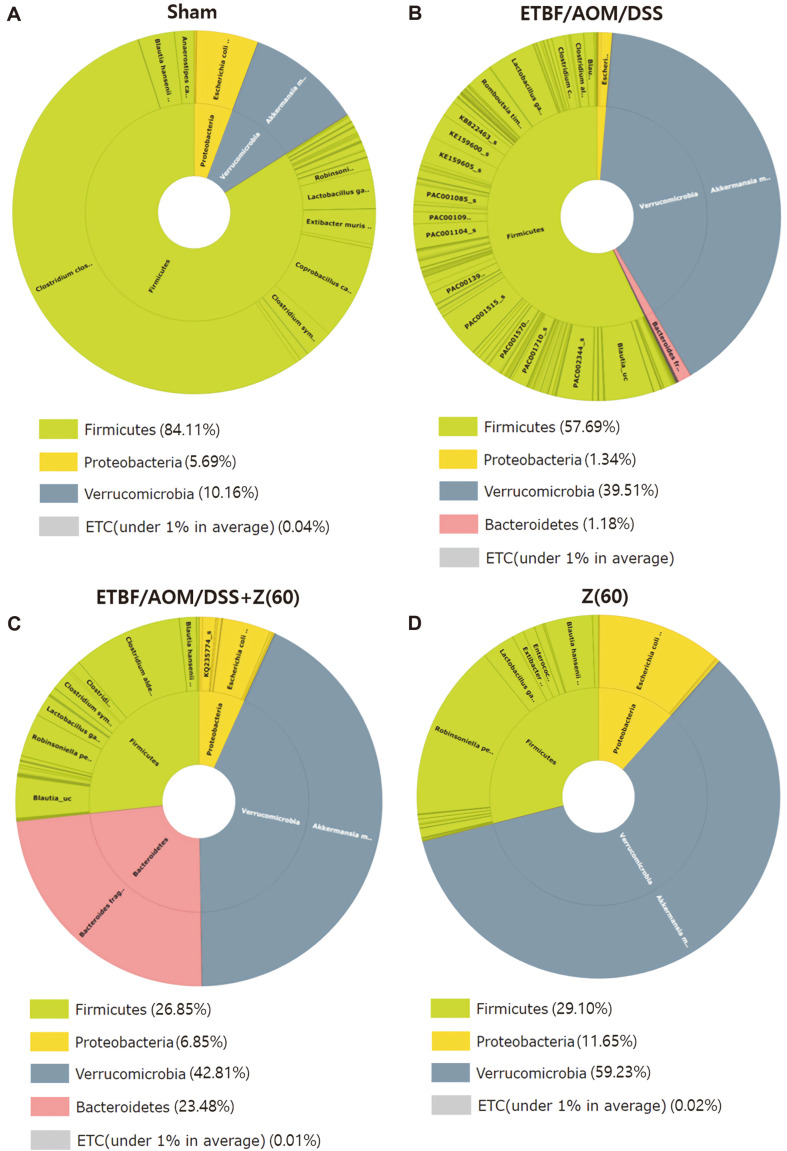
Bacterial community composition of cecum contents in each group. The relative abundances of 16S rRNA gene sequences (V3-V4 region), classified to the phylum (inner pie) and species (outer pie) level, are shown. Firmicutes, the phylum to which *Clostridium clostridioforme* group belongs was the major phylum in (**A**) sham control and (**B**) ETBFcolonized AOM/DSS group. Verrucomicrobia the phylum to which *Akkermansia muciniphila* belongs was the major phylum in (**C**) ETBF-colonized AOM/DSS group administered with 60 mg kg^-1^ zerumbone and (**D**) control group administered with 60 mg kg^-1^ zerumbone. Abbreviations of group names are as follows: *Sham*, sham control; *ETBF/AOM/DSS,* ETBF colonized AOM/DSS; *ETBF/AOM/DSS + Z(60),* ETBF colonized AOM/DSS given zerumbone (60 mg kg^-1^); Z (60), zerumbone (60 mg kg^-1^).

Taxonomic composition data at the level of phylum showed that three phyla were dominant, including Firmicutes (84.11%), Verrucomicrobia (10.16%), Proteobacteria (5.69%) in sham group (Fig. 1A). Of these major phyla in fecal microbiota of the sham group, Firmicutes (84.11%) was the most predominant phylum. Proportions of Verrumicrobia in ETBF colonized AOM/DSS, ETBF/AOM/DSS + Z (60), and zerumbone (60 mg kg^-1^) groups were increased (39.51%, 42.81%, 59.23%, respectively) (Figs. 1B-1D). In addition, the level of *Bacteroides* was higher in the ETBF/AOM/DSS + Z (60) group than that in ETBF colonized AOM/DSS group (23.48% and 1.18%, respectively, *p*-value: 0.00149). Results indicated a microbial diversity was influenced and more balanced in ETBF/AOM/DSS + Z (60) compared with ETBF-colonized AOM/DSS group (Fig. 1).

### Gut Microbiota Abundance at Family Level

**Fig. 2:**
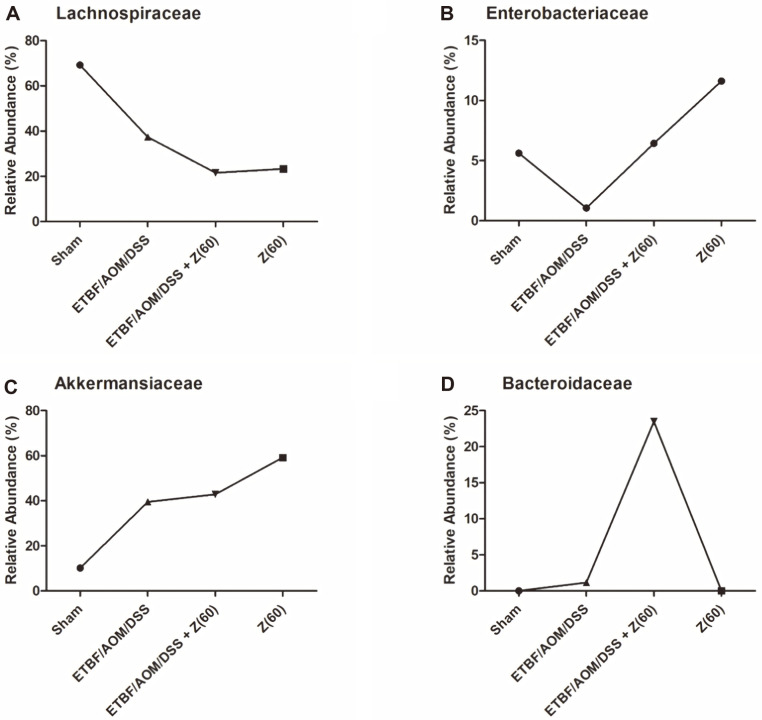
Gut microbiota at family level (*Lachnospiraceae*, *Akkermansiaceae*, *Enterobactericeae*, *Bacteroidaceae*). Significant families of each group based on 16S rRNA sequencing. Abbreviations of group names are as follows: *Sham*, sham control; *ETBF/AOM/DSS,* ETBF colonized AOM/DSS; *ETBF/AOM/DSS + Z(60),* ETBF colonized AOM/DSS given zerumbone (60 mg kg^-1^); Z (60), zerumbone (60 mg kg^-1^).

Average taxonomic compositions of MTP sets were also determined at family level (Fig. 2). We analyzed and focused on four families (*Lachnospiraceae*, *Enterobactericeae*, *Akkermansiaceae*, *Bacteroidaceae*) seen as the most predominant families in overall group. Compared to other groups, families that were the most different from sham group were *Lacchnospiraceae* and *Akkermansiaceae*. While *Lacchnospiraceae* decreased greatly, *Akkermansiaceae* showed an obvious increase (Figs. 2A and 2C). *Enterobacteriaceae* was significantly decreased in ETBF colonized AOM/DSS group. It was found to have high relative abundance as much as in the sham group and in ETBF-colonized AOM/DSS mice given zerumbone (Fig. 2B). *Bacteroidaceae* was significantly more abundant in the gut microbiota of ETBF/AOM/DSS + Z (60) group than that of ETBF colonized AOM/DSS group (Fig. 2D). Interestingly, results showed that phylum Bacteroidetes was not detected in sham or zerumbone (60 mg kg^-1^) group, although it was detected at low portion in the ETBF colonized AOM/DSS group and increased in the ETBF/AOM/DSS + Z (60) group at phylum level. Similarly, family *Bacteroidaceae* was increased in the ETBF/AOM/DSS+ Z (60) group.

### Microbial Compositions for Each Group at Genus and Species Levels

**Fig. 3:**
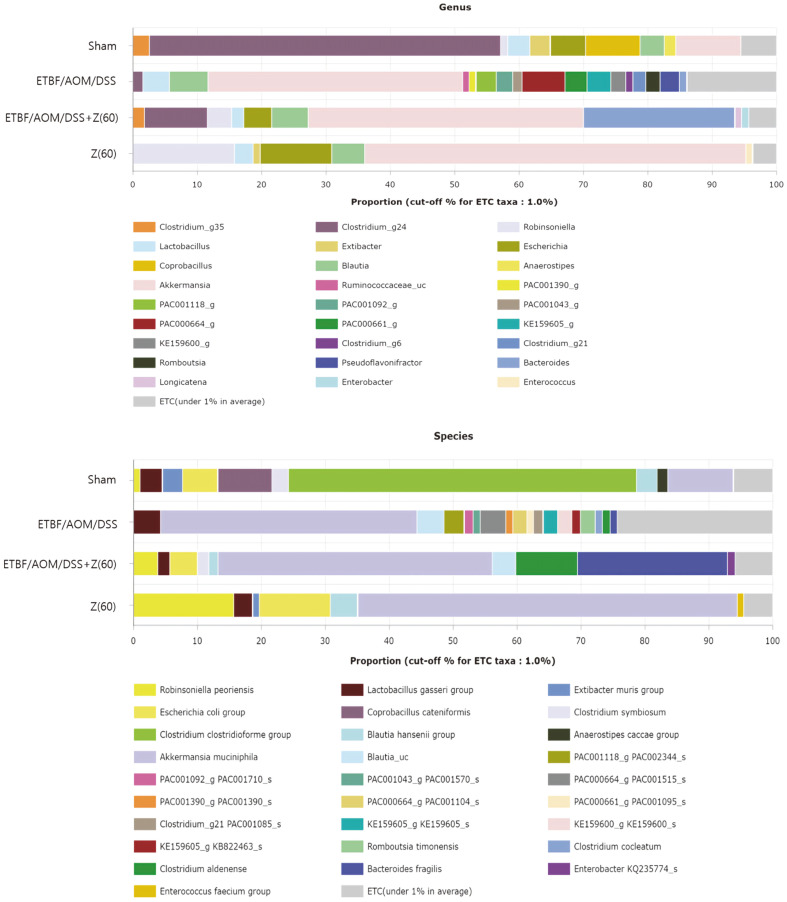
The proportion of gut microbiome at genus and species levels in each group. (**A**) Proportion of gut microbiome at genus level. (**B**) Averaged taxonomic compositions at species levels. Nomenclature is based on the EzTaxon-e database. Abbreviations of group names are as follows: *Sham*, sham control; *ETBF/AOM/DSS,* ETBF colonized AOM/DSS; *ETBF/AOM/DSS + Z(60),* ETBF colonized AOM/DSS given zerumbone (60 mg kg^-1^); Z (60), zerumbone (60 mg kg^-1^).

To further determine microbiome populations at genus level, relatively balanced microbial composition was observed in the ETBF/AOM/DSS + Z (60) group during the experiment (Fig. 3A). *Akkermansia* rapidly increased in the zerumbone (60 mg kg^-1^) group (59.23%). We observed a relative abundance of *Clostridium_g24* in sham group (54.64%). It was significantly reduced in the ETBF colonized AOM/DSS group to 1.57%. It was then enriched after given zerumbone (9.74%). Like the phylum level, the proportion of *Bacteroides* was higher in the ETBF/AOM/DSS + Z (60) group (23.49%) than that in the ETBF colonized AOM/DSS group (1.16%). Compared to other groups, various strains were seen in ETBF colonized AOM/DSS group. In addition, the ETC (under 1% in average) was significantly increased (13.92%).

In order to investigate changes in gut microbiota in detail, averaged taxonomic compositions of each group were analyzed at species levels based on the EzTaxon-e database [33]. In ETBF-colonized AOM/DSS group, the most abundant species was *Akkermansia muciniphila* (40.11%), followed by *Blautia_uc* (4.28%), and *Lactobacillus gasseri* (4.19%). On the other hand, the most abundant species was *Akkermansia muciniphila* (42.88%), followed by *Bacteroides fragilis* (23.52%), and *Clostridium aldenense* (9.64%) in ETBF/AOM/DSS + Z (60) (Fig. 3B). It was apparent that zerumbone-induced interventions affecting the composition of the intestinal microbiota may be a strategy to prevent the microbial dysbiosis prompted by ETBF.

### Taxonomic Biomarker Discovery Based on LEfSe Analysis of Gut Microbiota among Four Groups

**Fig. 4:**
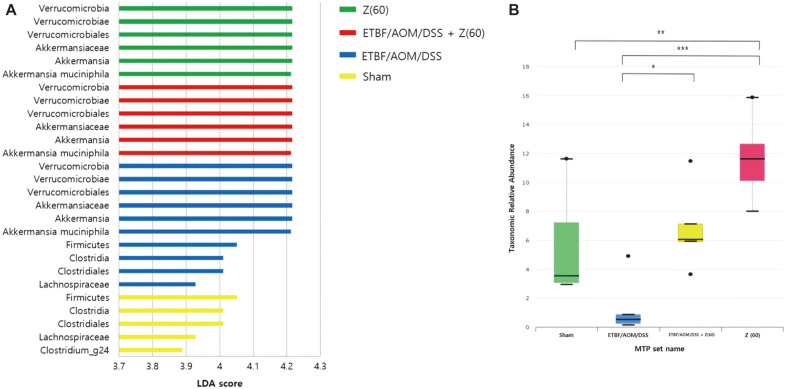
LEfSe analysis of different abundance levels. (**A**) Histogram of LDA scores for different abundance levels. LDA scores represent the effect size of each abundance level. Levels enriched in each group with an LDA score >2 are considered. (**B**) Taxonomic relative abundant box plot of Proteobacteria. LDA score was calculated using LEfSe, a metagenome analysis approach. Abbreviations of group names are as follows: *Sham*, sham control; *ETBF/AOM/DSS,* ETBF colonized AOM/DSS; *ETBF/AOM/DSS + Z(60),* ETBF colonized AOM/DSS given zerumbone (60 mg kg^-1^); Z (60), zerumbone (60 mg kg^-1^).

We also performed LEfSe analysis at various level to assess which microbiota were driving divergence between different groups using parameters described above (Fig. 4). LEfSe analysis revealed that not only phylum Verrucomicrobia and its derivative class Verrucimicrobiae, order Verrucomicrobiales, but also family *Akkermansiaceae* were significantly higher in the zerumbone (60 mg kg^-1^) group and ETBF/AOM/DSS + Z (60) group, but significantly lower in the intestinal microbiota from sham group (LDA score: 4.216, *p*-value: 0.00423). Additionally, LEfSe analysis revealed that phylum Firmicutes (LDA score: 4.051, *p*-value: 0.00168), class Clostridia and order Clostridiales (LDA score: 4.01, *p*-value: 0.00182) were significantly higher in sham and ETBF colonized AOM/DSS groups. Especially, *Clostridium_g24* genus was significantly higher in the sham group (LDA score: 3.890, *p*-value: 0.00089).

### Alpha Diversity of Gut Microbiota in Each Group

**Fig. 5:**
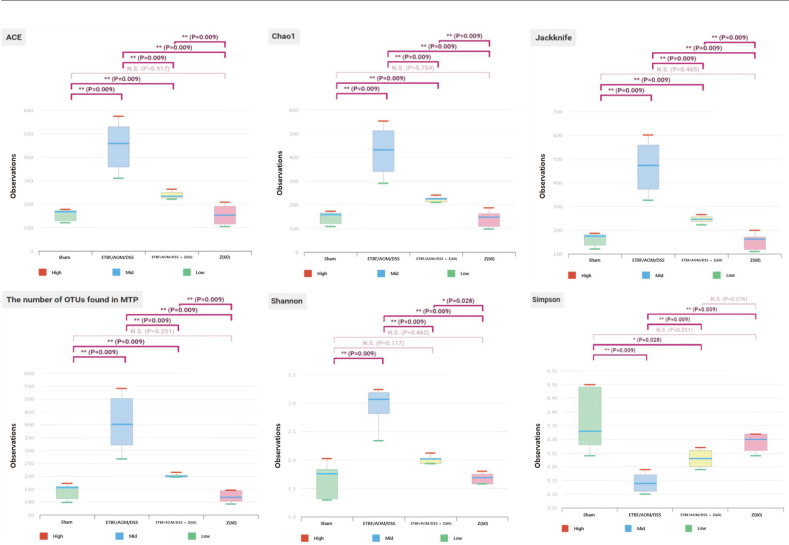
Alpha-diversity calculated using phylotype relative abundance measurrements in each group. Box plot shows similar aspest among each group. ETBF/AOM/DSS group tended to be higher than other groups. We measured ACE, Chao1, Jackknife, the number of OTUs found in MTP, Shannon and Simpson index based on 16S rRNA gene sequencing data. Abbreviations of group names are as follows: *Sham*, sham control; *ETBF/AOM/DSS,* ETBF colonized AOM/DSS; *ETBF Z(60),* ETBF colonized AOM/DSS given zerumbone (60 mg kg^-1^); Z, zerumbone (60 mg kg^-1^).

We compared alpha diversity between groups. Alpha diversity is known to reflect the richness and evenness by measuring the number of OTUs found in MTP, Shannon diversity index etc. based on 16S rRNA gene sequencing data. Alpha diversity evaluation among the four groups using six different metrics are shown in Fig. 5. We used values given by one or more diversity index such as species richness (ACE, Chao 1, Jackknife, number of OTUs found in MTP) and diversity index (Shannon index or the Simpson index). Overall, alpha diversity scores tended to be higher (except the Simpson index) for the ETBF colonized AOM/DSS group but lower for the sham group, ETBF/AOM/DSS + Z (60) group, and zerumbone (60 mg kg^-1^) group. OTU measurements and Shannon Index of ETBF colonized AOM/DSS group were significantly increased, demonstrating that there was richness and various strains. Whereas in zerumbone group and sham group, the diversity decreased significantly. The ETBF/AOM/DSS + Z (60) group showed a tendency to be similar to sham group. These results indicate that zerumbone can restore changes induced by AOM/DSS treatment to decrease/increase the abundance of these microbial species so that they can resemble the sham group.

### Assessment of Group Similarities in Species Composition by Beta Diversity

**Fig. 6:**
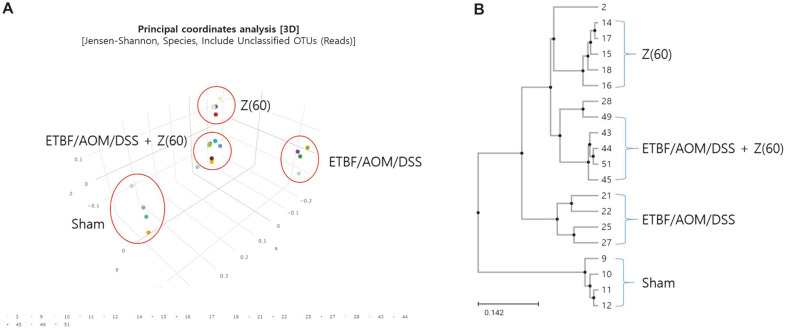
Assessment of group similarities in species composition by beta diversity. (**A**) 3D principal coordinates analysis (PCoA) of Sham, ETBF, ETBF + Z (60), Z (60). Each point based on principal component score 1, 2, and 3 of all samples in a group. (**B**) UPGMA dendrogram. Each sample was clustered by subject. Abbreviations of group names are as follows: *Sham*, sham control; *ETBF/AOM/DSS,* ETBF colonized AOM/DSS; *ETBF/AOM/DSS + Z(60),* ETBF colonized AOM/ DSS given zerumbone (60 mg kg^-1^); Z (60), zerumbone (60 mg kg^-1^).

Beta diversity was assessed using principal coordinates analysis (PCoA) scores and UPGMA dendrogram (Fig. 6). PCoA based on the relative abundance of between groups of samples revealed that a significant separation in bacterial community composition using the first three principal coordinates scores of PC1, PC2, and PC3. The gut microbiota of each group was significantly different from that of other groups based on both PCoA scores plot and UPGMA dendrogram. In addition, we could identify each sample was clustered by subject. PCoA and dendrogram showed almost the same results. From the result that zerumbone (60 mg kg^-1^) group and ETBF/AOM/DSS + Z (60) group were relatively close in PCoA and that they were almost on one plane, it could be concluded that compositions of strains between groups were similar (Fig. 6A). The only zerumbone-treated group indicated distinct position compared to sham control in PCoA score, suggesting that zerumbone treatment changes microbial community with or without inflammation or tumorigenesis. In dendrogram, sham group seemed to have a different cluster from that of other groups based on Fast Unifrac distance (Fig. 6B). Beta diversity results based on PCoA and dendrogram demonstrated similarities in bacterial community structure among ETBF/AOM/DSS + Z (60) group and zerumbone group.

### Assessment of Firmicutes to Bacteroidetes Ratio (F/B Ratio)

**Fig. 7:**
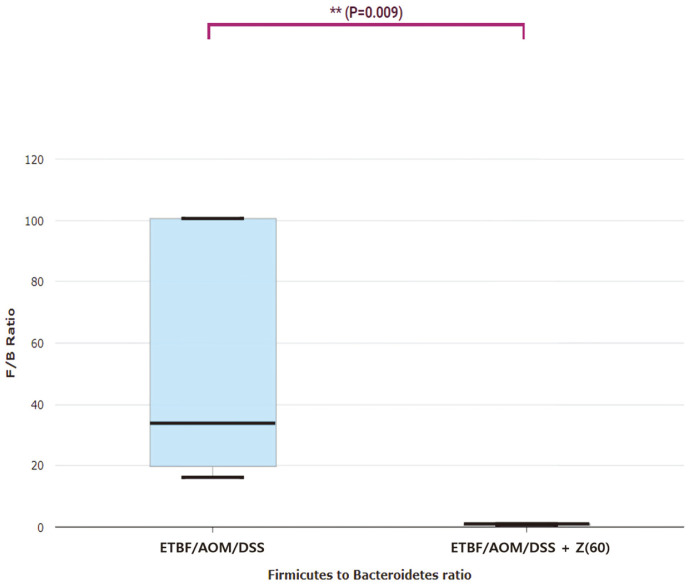
Firmicutes to Bacteroidetes ratio (F/B ratio). F/B ratio of ETBF colonized AOM/DSS and ETBF colonized AOM/DSS given zerumbone (60 mg kg^-1^). The F/B ratio of ETBF colonized AOM/DSS was significantly higher than that of ETBF/AOM/DSS + Z (60) group. Abbreviations of group names are as follows: *ETBF/AOM/DSS,* ETBF colonized AOM/DSS; *ETB/AOM/DSS + Z(60),* ETBF colonized AOM/DSS given Zerumbone (60 mg kg^-1^).

We analyzed the F/B ratio with given result between ETBF/AOM/DSS + Z (60) group and ETBF colonized AOM/DSS group (Fig. 7). The F/B ratio of the ETBF colonized AOM/DSS group was much higher than that of the ETBF colonized AOM/DSS given zerumbone. This means that there is a bowel disease in the ETBF colonized AOM/DSS group.

## Discussion

In this study, to know how fecal microbiota might change after given zerumbone, we first analyzed compositions of gut microbiota and determined whether zerumbone could restore gut microbiota of ETBF-colonized AOM/DSS group. Our results revealed significant difference of intestinal microbiota composition between ETBF/AOM/DSS + Z (60) group and ETBF colonized AOM/DSS group. The relative abundance of dominant phyla Firmicutes, Proteobacteria, Verrucomicrobia, *Bacteroides* (Fig. 1), and dominant families *Lachnospiraceae*, *Enterobactericeae*, *Akkermansiaceae*, *Bacteroidaceae* were found (Fig. 2).

Firmicutes is one of the dominant phyla within healthy adult microbiota. A quantitative reduction of this phylum has been shown in inflammatory gut disease [39]. Additionally, as observed in humans, python gut, and a wide variety of other mammals, most sequences were classified as Firmicutes (61.8%) [40, 41]. Our results showed that phylum Firmicutes was the most abundant in the sham group while it was the least abundant in the two zerumbone treated groups (ETBF/AOM/DSS + Z (60) and zerumbone). The ETBF colonized AOM/DSS group had higher abundance of Firmicutes than the groups given zerumbone. A previous study has shown that healthy intestinal microbiota is predominantly constituted by phyla Firmicutes and Bacteroidetes [42]. Referred to contents of the previous study, microbiome detection in sham group is predicted to be normal flora. After processing ETBF/AOM/DSS, the formation of CRC resulted in a significantly decrease in Firmicutes and zerumbone treatment showed reduction in the number of species involved in inflammatory reaction.

Microbiome taxonomic profiling (MTP) results showed that microbial dysbiosis was improved in ETBF/AOM/DSS + Z (60). The second highest species of gut microbes in the cecum of ETBF/AOM/DSS + Z (60) was *Bacteroides fragilis* (Fig. 3) compared to other groups. A recent study showed that non-toxigenic *Bacteroides fragilis* (NTBF), a microbe of the normal microbiota of the human colonic commensal [43], diminished ETBF-induced colitis and colitis-promoted tumorigenesis in Min mice [44]. Collectively, these results suggest that increased NTBF by oral treatment of zerumbone may tamper ETBF-promoted tumorigenesis in AOM/DSS mice.

It is definite that NTBF and ETBF cannot be classified by only 300-bp 16S rRNA gene fragments. To distinguish the NTBF and ETBF, we took phenotypic test in previous study [45, 46]. We used *B. fragilis* 86-5443-2-2 (*bft-2*) as ETBF strain in the current study, which is resistant to gentamicin and clindamycin. Mouse stool was monitored by serial dilution and inoculating of stool on brain heart infusion agar (BHIA) plates including 50 μg/ml of gentamicin and 6 μg/ml of clindamycin. Plates were incubated overnight at 37oC under anaerobic conditions. Other fecal bacteria including NTBF were prevented by addition of gentamicin and clindamycin. Characteristic ETBF colonies were enumerated after anaerobic culture and shown as colony-forming units (CFU)/g stool.

Proteobacteria known to be less abundant in CRC patients are generally regarded as intestinal normal flora with potential-pathogenic features [47]. Our results showed a similar pattern to the previous research. The proportion of Proteobacteria in the ETBF colonized AOM/DSS group was the lowest (1.34%). It was the most abundant in the zerumbone-treated group and the other two groups (sham and ETBF/AOM/DSS + Z (60)) were almost similar proportion. In addition, referring to LEfSe analysis, the microbial diversity of Proteobacteria dropped significantly in the ETBF/AOM/DSS group. However, after given zerumbone, the taxonomic relative abundance was increased to be as much as sham (Fig. 4). These results demonstrate that zerumbone could restore compositions of intestinal microbiota in mice with CRC caused by ETBF.

In mice and a wide variety of other mammals, most gut microbiome sequences were detected as either Firmicutes (61.8%) or Bacteroidetes (20.6%) [41]. According to a recent study, normal human gut microbiota comprises of two major phyla, namely Bacteroidetes and Firmicutes [42]. In addition, Chan *et al*. have found that non-toxigenic *B. fragilis* (NTBF) can mitigate colitis and tumorigenesis initiated by ETBF as long as NTBF introduction precedes ETBF strain exposure and remains the dominant *B. fragilis* strain [44]. In our study, Bacteroidetes was more abundant in the ETBF/AOM/DSS + Z (60) than ETBF colonized AOM/DSS. Based on these results, we could conclude that increase in Bacteroidetes of ETBF/AOM/DSS + Z (60) group reflects an effect of NTBF by oral treatment.

One study has found that *Lactobacillus salivarius* strain UCC118 belonging to phylum Firmicutes can produce a two-component bacteriocin against bacteria of Bacteroidetes [48]. In addition, Guinane *et al*. revealed that bactofencin A has a potentially positive influence on both intestinal communities and anaerobic microbiome such as *Bacteroides* [49]. The ETBF/AOM/DSS + Z (60) group showed many normal strains of Bacteroidetes. It might be attributed to decreased production of bacteriocin in Firmicutes.

The Firmicutes to Bacteroidetes ratio (F/B ratio) has been used as an indicator of disease and extensively examined for human and mouse gut microbiota [50]. Also, cooperation between Firmicutes and Bacteroidetes in the body mass index has been reported [51]. An increase in Firmicutes characterizes obesity [52]. An increase in Bacteroidetes has been reported to occur in those aged 30 years and older [53]. Many studies have shown that the F/B ratio is correlated with diseases. In our study, the F/B ratio of the ETBF/AOM/DSS + Z (60) group was almost 1 (Fig. 7). Therefore, this potentially means that zerumbone can regulate Firmicutes/Bacteroides composition and consequently improve intestinal microbial dysbiosis by balancing.

In previous studies demonstrated that zerumbone prevented both biofilm formation and biofilms pre-formed by ETBF [54], and reported that zerumbone has anti-inflammatory effect of ETBF infection without affecting ETBF colonization [45, 46]. However, further replication studies, to clarify the prevention and to examine the balancing effects of zerumbone in the pharmaceutical field, should follow in a near future.

In conclusion, our study showed increased diversity and richness in ETBF/AOM/DSS + Z (60) compared with ETBF colonized AOM/DSS group through a number of analytical parameters, including alpha or beta diversity. Additionally, our results significantly indicated that zerumbone can ease intestinal microbial imbalance against ETBF and increase intestinal microbial diversity. Therefore, zerumbone is proposed as a promising natural preventive agent along with conventional antimicrobial agents against ETBF-associated colorectal cancer with the potential to maintain human health via dietary supplementation.
